# Maturation and development of fetal pig intestinal tissue in immunodeficient mice

**DOI:** 10.1590/acb390624

**Published:** 2024-02-26

**Authors:** Atsushi Harada, Naoto Matsumoto, Yoshitaka Kinoshita, Kenji Matsu, Yuka Inage, Keita Morimoto, Shuichiro Yamanaka, Masashi Kurobe, Takashi Yokoo, Haruki Kume, Takao Ohki, Eiji Kobayashi

**Affiliations:** 1 The Jikei University School of Medicine – Division of Pediatric Surgery – Department of Surgery – Tokyo – Japan.; 2 The Jikei University School of Medicine – Division of Nephrology and Hypertension – Department of Internal Medicine – Tokyo – Japan.; 3 The University of Tokyo – Graduate School of Medicine – Department of Urology – Tokyo – Japan.; 4 The Jikei University School of Medicine – Department of Kidney Regenerative Medicine – Tokyo – Japan.; 5 The Jikei University School of Medicine – Department of Pediatrics – Tokyo – Japan.

**Keywords:** Fetal Organ Maturity, Transplantation, Heterologous, Enteric Nervous System, Organoids

## Abstract

**Purpose:**

This study aimed to compare the degree of maturation and development of fetal pig segmental intestinal tissue with that of spheroids created by in-vitro reaggregation of dissociated fetal intestinal cells after transplantation into immunodeficient mice.

**Methods:**

Fetal pig small intestines were transplanted as segmental grafts into the omentum and subrenal capsules of immunodeficient mice or enzymatically treated to generate single cells. Spheroids made by in-vitro reaggregation of these cells were transplanted into the subrenal capsules of immunodeficient mice. The segmental grafts and spheroids were harvested four and eight weeks after transplantation, and the structural maturity and in-vivo development of these specimens were histologically evaluated.

**Results:**

The spheroids were engrafted and supplied blood vessels from the host mice, but an intestinal layered structure was not clearly observed, and there was almost no change in size. On the other hand, the segmental grafts formed deep crypts in the mucus membrane, the inner circular layer, and outer longitudinal muscles. The crypts of the transplanted grafts harvested at eight weeks were much deeper, and the smooth muscle layer and the enteric nervous system were more mature than those of grafts harvested at the fourth week, although the intestinal peristaltic wave was not observed.

**Conclusions:**

Spheroids created from fetal small intestinal cells could not form layered structures or mature sufficiently. Conversely, segmental tissues structurally matured and developed after in-vivo transplantation and are therefore potential grafts for transplantation.

## Introduction

Recent advances in gene modification techniques such as CRISPR–Cas9 have brought renewed attention to pigs as sources for xenotransplantation^1^^,^ 2. However, several issues remain, such as the excessive immunosuppression required in recipients and infectious diseases that may be introduced in the subject, as observed in fatal cases of heart transplantation in genetically modified pigs that were created with the latest technology^3^. Meanwhile, it is believed that the immunological advantage of fetal pig organs during development can potentially reduce the doses of immunosuppressants after transplantation in humans. In the early stage of xenotransplantation therapy, Groth et al. succeeded in transplanting pig islets to humans via xenotransplantation and revealed that pig pancreatic endocrine tissue can survive in the human body^4^.

We have also considered using this immunological advantage of fetal pig donors^5^ and compared the extent of rejection of a fetal pig kidney transplanted into adipose tissue and a newborn pig kidney transplanted with anastomosis of its blood vessels in a recipient monkey. The results showed that the former was sufficiently controlled with immunosuppressive drugs that are used in human clinical practice^6^.

Recently, research on human intestinal organoids (HIOs) derived from pluripotent stem cells (PSCs) has progressed remarkably. As PSCs potentially differentiate and develop into intestinal organoids containing most of the cell types of the intestine in the proper conditions, organoids are also expected as the cell sources of intestinal transplantation. However, there are still problems; organoids do not exhibit the complete function of the intestine, as current methods do not reproduce the functional coordination between each organization differentiated from all germ layers that occur during in-vivo organogenesis.

The current study was performed to compare the degree of in-vivo maturation after transplantation of spheroids created through reaggregation of dissociated fetal intestinal cells and fetal segmental tissue in order to assess the potential of fetal pigs as tissue and cell sources for small intestinal regeneration. We examined the maturation of fetal pig intestinal tissue transplanted into omental tissue and the renal subcapsular region in immunodeficient mice. Additionally, we investigated angiogenesis into the transplanted graft and maturation of the enteric nervous system, such as Auerbach’s plexus and ganglion cells.

## Methods

### Experimental animals and ethics

The experiments were approved by the Ethics Committee of The Jikei University School of Medicine (approval number 2022-044). The experiments were conducted in conformance with the Guidelines for the Appropriate Conduct of Animal Experiments (2006) of the Science Council of Japan^7^.

### Transplantation of the intestines of fetal pigs into immunodeficient mice

Fetuses at embryonic day 30 (E30) obtained from pregnant microminipigs via cesarean section were purchased from Fuji Micra Inc. (Shizuoka, Japan) and used as donors of fetal intestinal grafts. Eight-week-old male NOD/ShiJic-scid Jcl mice purchased from CLEA Japan, Inc. (Tokyo, Japan) were used as recipients (spheroid transplantation: N = 2, segmental intestinal transplantation: N = 8).

*Transplantation of the fetal intestines of B6 mice into adult B6 mice*

Fetuses (E13) were obtained from pregnant C57BL/6JJmsSlc mice purchased from Sankyo Labo Service Corporation, Inc. (Tokyo, Japan) and used as donors of fetal intestinal grafts. Seven-week-old C57BL/6JJmsSlc male mice (N = 3) were used as recipients for syngeneic transplantation as controls.

### Anesthesia and euthanasia methods

#### Procurement of small intestines from fetal pigs and B6 mice as grafts for transplantation

Pregnant pigs were fasted for 12 hours before surgery but allowed to drink water. After sedation via an intramuscular injection of 2-mg/kg xylazine, anesthesia was induced with inhaled 5% isoflurane and maintained at 1–3%. E30 fetal pigs were delivered by cesarean section via a 10-cm midline incision; the incision of the pregnant pig was closed after delivery. The fetuses were immediately euthanized by decapitation, and the fetal intestine was procured under a surgical microscope. Pregnant B6 mice (E13) were also maintained under anesthesia at 1–3% isoflurane after induction with 5% isoflurane. After cesarean section, the pregnant mice were euthanized by exsanguination. The fetuses were also immediately euthanized by decapitation, and the fetal intestine was procured under a surgical microscope.

#### Recipient surgery

After induction with 5% isoflurane, the recipient mice underwent transplant procedures under 1–3% isoflurane maintenance anesthesia and were kept warm until recovery.

#### Care and observation of recipient animals

Experimental animals were given water and food ad libitum in an environment with a 12-h light-dark cycle. The recipient mice were euthanized by cervical dislocation, and the transplanted tissues were obtained at the end of the study.

### Method of fetal intestinal transplantation

#### Fetal intestinal spheroid formation

The fetal intestinal tissues were collected in 1.5-mL tubes containing Gibco^TM^ minimum essential medium α (MEMα) (Thermo Scientific, Waltham, MA, United States of America). The tubes were centrifuged at 300 × g for 5 min, and the supernatant was removed. Then, 1,000 μL of Accutase^TM^ (Innovative Cell Technology, San Diego, CA, United States of America) was dispensed at room temperature and mixed by vortexing. The cell suspension was incubated for 5 min, pipetted, vortexed again, and centrifuged at 300 × g for 5 min, and the supernatant was removed. Then, 1,000 μL of medium containing 10 μM Y2763 (Wako, Osaka, Japan) in MEMα with 10% fetal bovine serum (FBS) was added, and the cell suspension was filtered through a 40-μm cell strainer (BD Falcon, Oxford, United Kingdom) to obtain a single-cell suspension. This single-cell suspension was adjusted to 1 × 10^6^ cells/mL with medium after the cells were counted. The cells were divided into U-bottom 96-well low-cell-binding plates (Thermo Scientific, Waltham, MA, United States of America), with an average of 2 × 10^5^ cells/200 μL/well. Finally, the cells were centrifuged at 1,000 rpm for 4 min and incubated at 37°C. The next day, the medium was changed to medium without Y2763; the medium was then changed once every two days. The spheroids were collected on the fifth day.

#### Spheroid and segmental intestinal tissue transplantation into the subrenal capsules of immunodeficient mice

Subrenal capsule transplantation was performed as described in our previous report^8^. Briefly, under general anesthesia, laparotomy was performed by creation of a midline incision in the recipient NOD/ShiJic-scid Jcl mouse. A space in the subrenal capsule was created in the lower pole of the kidney using water pressure, and spheroids and approximately 3 mm of segmental fetal small intestine were transplanted. Four weeks later, the specimens were collected and histologically evaluated.

#### Omental transplantation of segmental fetal small intestine

The fetal small intestine was cut into segments of 3 mm. An 8-0 nylon thread was passed through the lumen under a microscope. The omental transplantation method was performed as described in our previous report^9^. Briefly, after laparotomy via midline abdominal incision under general anesthesia, the fetal intestine was wrapped and rolled in the omentum. Then, the surgical incision was closed. Four and eight weeks later, the mice were euthanized, and the specimens were collected and histologically evaluated.

### Histological evaluation

Transplanted grafts were harvested after four and eight weeks and evaluated for structural maturity and development. Samples were fixed in 4% paraformaldehyde (Wako, Osaka, Japan), dehydrated in 20% sucrose, and embedded in optimum cutting temperature (OCT) compound (Sakura Finetek, Tokyo, Japan). Sections were prepared at a thickness of 8–10 μm. Hematoxylin and eosin staining and Alcian blue staining were performed using standard procedures for histological analysis.

#### Immunohistochemical staining

After these sections were washed for 5 minutes in phosphate-buffered saline (PBS: 0.01 mol/L, pH 7.2), they were processed for antigen retrieval by heating at 70°C in HistoVT One (Nacalai Tesque, Kyoto, Japan) for 20 min. After washing in PBS, a blocking agent, Blocking One Histo (Nacalai Tesque, Kyoto, Japan), was used to prevent nonspecific binding for 10 min, after which the sections were incubated with primary antibodies (1:1,000) for 24 hours at 4°C. Primary antibodies against αSMA (Abcam, Cambridge, United Kingdom), CD31 (R&D Systems, MN, United States of America), and E-cadherin (BD Biosciences, Tokyo, Japan) were used. After washing three times with PBS, the samples were incubated with 1:500 dilutions of secondary antibodies conjugated with Alexa Fluor 488, 555, and 647 for 1 h at room temperature. The samples were washed three times in PBS and mounted in mounting medium plus 4’,6-diamidino-2-phenylindole (DAPI) (Thermo Fisher Scientific, Waltham, MA, United States of America). Additionally, the distribution of S-100 protein in the enteric nervous system was evaluated. Samples were stained with primary antibodies against S-100 (Leica IHC Refine Kit S-100, Leica Biosystems, Wetzlar, Germany) for 15 minutes. Next, rabbit anti-mouse IgG (BOND Polymer Refine Detection Kit, Leica Biosystems, Wetzlar, Germany) was applied for 10 minutes and then subsequently detected with anti-rabbit polymeric horseradish peroxidase (BOND Polymer Refine Detection Kit, Leica Biosystems, Wetzlar, Germany). Finally, the sections were incubated with 3,3-diaminobenzidine (DAB) and counterstained with hematoxylin. The sections were imaged with an all-in-one fluorescence microscope (BZ-X800, Keyence, Osaka, Japan) and a confocal microscope (LSM 880, Carl Zeiss, Munich, Germany).

## Results

### Spheroid formation and transplantation into the subrenal capsule

**Figure 1:**
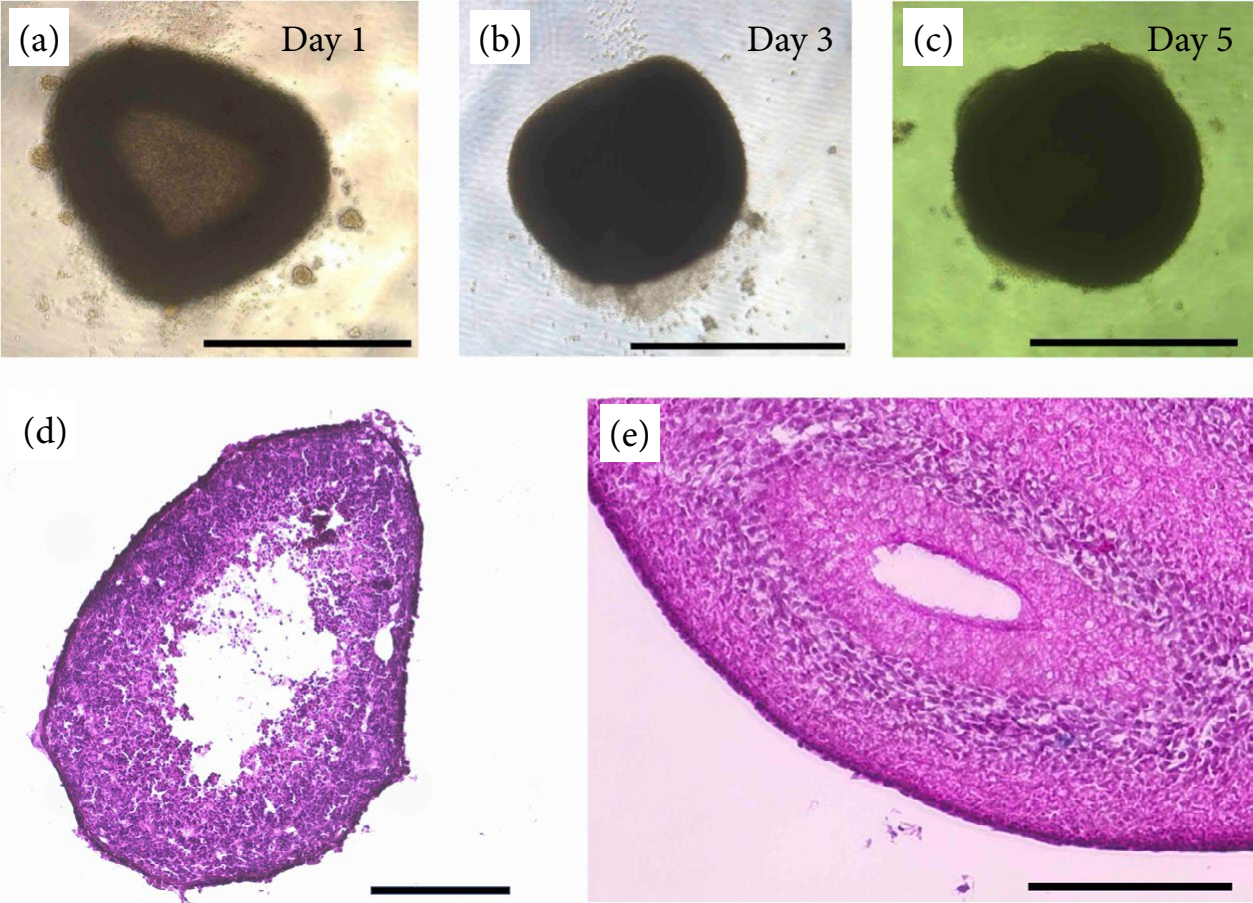
Time course of reaggregation of spheroids from fetal pig intestines in vitro.

Source: Elaborated by the authors.

Spheroids formed on day 1 of in-vitro culture. Histological evaluation of the spheroids on day 5 revealed that each spheroid was covered with an outer membrane and that the cells were aggregated into spheres (Figs. 1a–1c). However, a layered structure with a mucous membrane and mesenchymal layer was not confirmed compared to that in the fetal pig intestine (E30) without dissociation and reaggregation (Figs. 1d and 1e).

**Figure 2:**
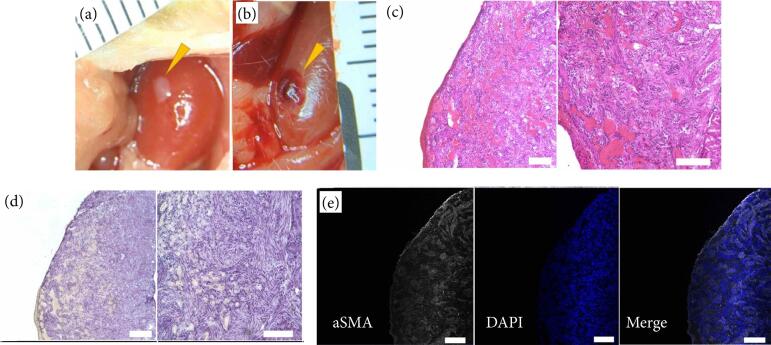
Histological findings of spheroids from fetal pig intestines four weeks after transplantation into the subrenal capsules of immunodeficient mice. The structures of the mucous membrane and mesenchymal layer were not clearly visible.

Source: Elaborated by the authors.

The spheroids were transplanted into the subrenal capsule and collected four weeks later. Although the spheroids, which were macroscopically white just after transplantation, turned reddish due to angiogenesis, there was almost no change in size (Figs. 2a and 2b). In histological analysis, the structure of the mucous membrane and mesenchymal layer was not confirmed by hematoxylin-eosin staining (Fig. 2c). In addition, Alcian blue-positive mucosal goblet cells could not be confirmed (Fig. 2d), although αSMA-positive smooth muscle cells were confirmed (Fig. 2e). In summary, although the spheroids were engrafted and supplied with blood, histological maturation and development were not observed.

### Transplantation of fetal pig segmental small intestinal tissue

#### Subrenal capsular transplantation in immunodeficient mice

**Figure 3:**
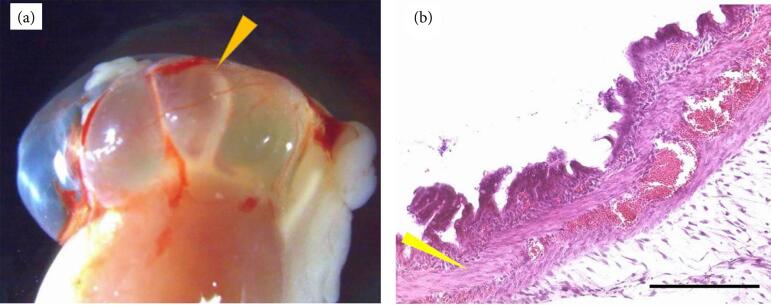
Findings obtained with segmental fetal pig intestines transplanted into the subrenal capsules of immunodeficient mice.

Source: Elaborated by the authors.

Segmental fetal intestinal tissues of pigs were harvested four weeks after subrenal capsular transplantation. Macroscopically, they were engrafted, supplied with blood vessels and partially dilated, with cyst formation (Fig. 3a). No obvious peristalsis was observed in the grafted fetal intestine. Histological analysis revealed the formation of deep crypts, and the inner and outer longitudinal muscles were visible compared with those of the E30 fetal small intestine before transplantation (Figs. 1e and 3b).

#### Omental transplantation in immunodeficient mice

**Figure 4:**
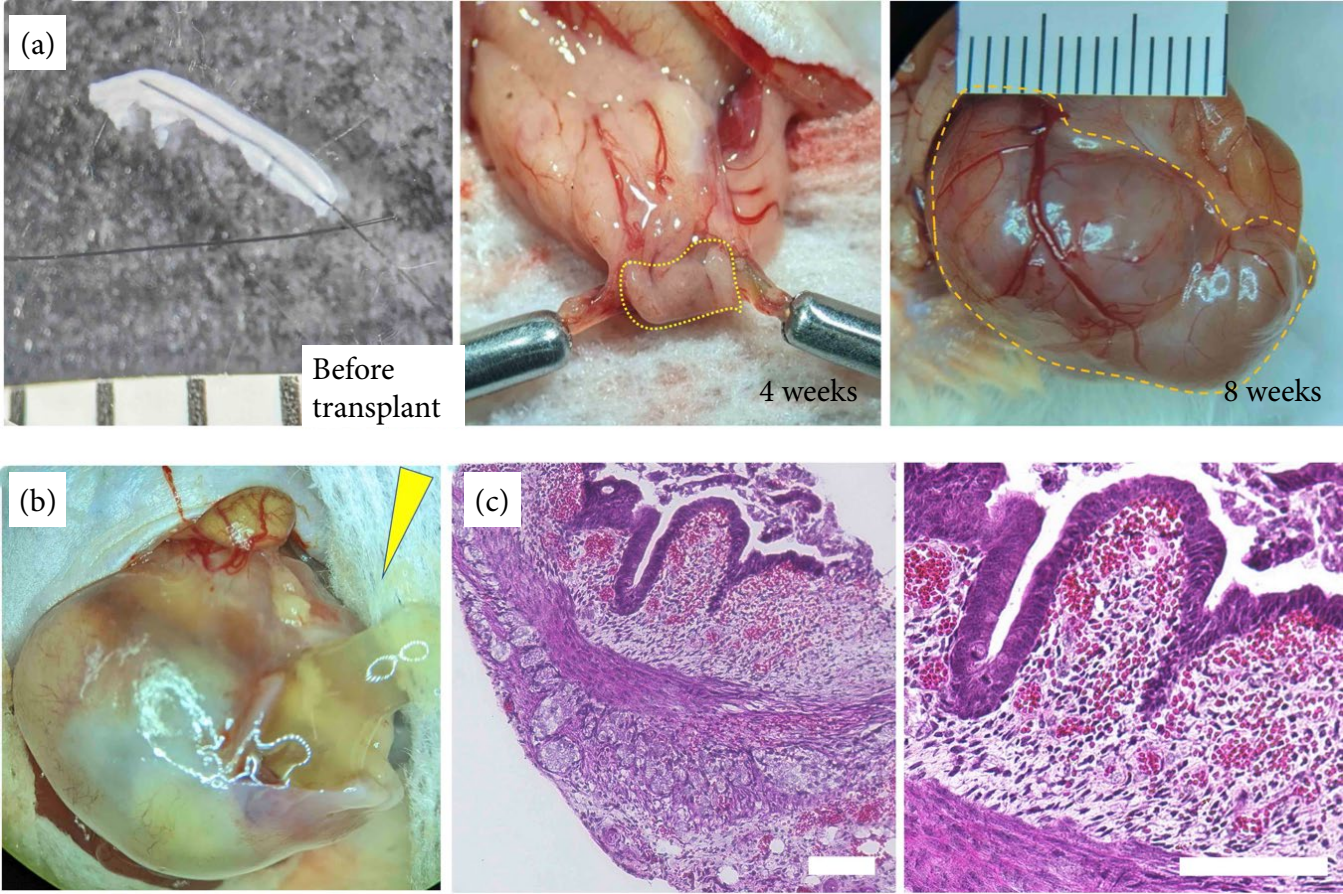
Time course of fetal pig intestines after omental transplantation in vivo.

Source: Elaborated by the authors.

Fetal intestinal grafts were harvested four and eight weeks after omental transplantation in immunodeficient mice. Their maximum diameter increased from 3 mm at the beginning to 5 mm at the fourth week and to 23 mm at the eighth week (Fig. 4a). Mucus-like secretions were observed in the developed grafts at eigth weeks (Fig. 4b), but obvious peristalsis was not observed in any grafts.

**Figure 5:**
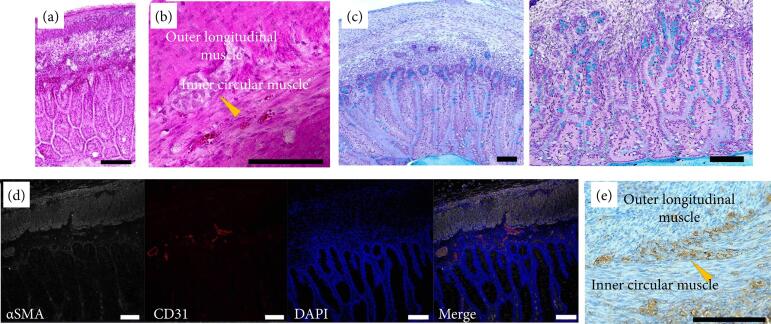
Histological findings of fetal pig intestines at eight weeks after transplantation into the omentum in immunodeficient mice.

Source: Elaborated by the authors.

Histological analysis revealed that the inner ring and outer longitudinal muscles were differentiated at both four and eight weeks (Fig. 4c: four weeks, Fig. 5a: eight weeks), and the formation of deep crypts and staining of goblet cells with Alcian blue were revealed (Figs. 5b and 5c) at the eighth week after transplantation. The smooth muscle layer differentiated into a two-layered structure with an inner and outer longitudinal muscle stained with αSMA, as revealed by immunohistochemical staining (Fig. 5d). Additionally, the maturation and development of vessels were confirmed by the presence of CD-31-positive vascular endothelial cells in the submucosal layer (Fig. 5d).

The crypts of the transplanted grafts at the eighth week were much deeper than those at the fourth week. The smooth muscle layer differentiated into two layers, and a mature enteric nervous system, including ganglion cells, was observed at the eighth week, although the intestinal peristaltic wave was not observed. The presence of S-100-positive neuron fibers and enteric nerve plexus within the muscular layer was confirmed, and ganglion cells were also observed in Auerbach’s plexus (Fig. 5e) at the eighth week after transplantation.

#### Syngeneic omental transplantation in B6 mice

**Figure 6:**
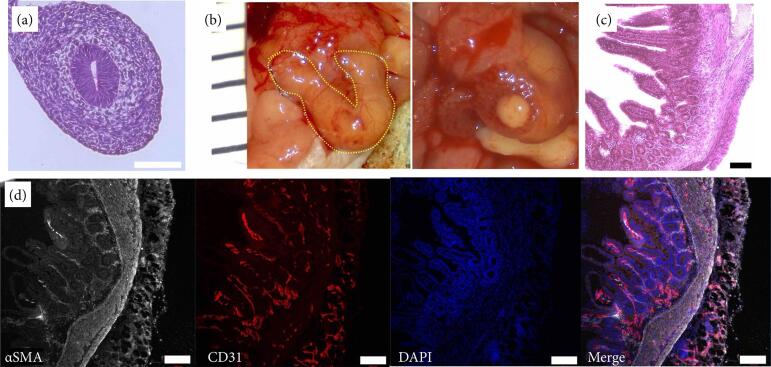
Histological findings of the fetal small intestine revealed maturation and development four weeks after syngeneic transplantation.

Source: Elaborated by the authors.

As a control, syngeneic transplantation was performed in C57BL/6JJmsSlc mice. Initially, fetal segmental small intestine (E13) specimens from C57BL/6JJmsSlc mice before transplantation were evaluated microscopically via hematoxylin-eosin staining and immunohistochemical staining. The specimens had a two-layer structure consisting of a mucous membrane and a mesenchymal layer (Fig. 6a). By the fourth week after omental transplantation, the size had increased from 3 to 9 mm, which was larger than the xenografted specimen at the same time point, and the patency of the lumen was confirmed (Fig. 6b). Histological analysis revealed deep crypts and differentiation of the muscle layer into a two-layered structure with an inner and outer longitudinal muscle (Fig. 6c). Immunohistochemical staining revealed αSMA-positive and CD31-positive cells, confirming the presence of intestinal smooth muscle and vascular endothelium, respectively, which indicated that the tissue was differentiated (Fig. 6d).

## Discussion

In this study, significant in-vivo maturation of the mucosal layer and muscle layer in the fetal segmental intestine was revealed. In contrast, the mucosal and muscle layers did not have a clear layered structure, and maturation was not observed in spheroid transplantation. This is the first report comparing maturation between fetal segmental intestinal tissue and fetal intestinal spheroids after xenotransplantation from pigs into mice.

Intestinal failure in childhood is a challenging and complicated condition resulting from inadequate small intestinal function in the setting of short bowel syndrome, such as in patients with the long-segment type of Hirschsprung’s disease or massive small intestine resection due to malrotation volvulus or necrotic enterocolitis. Intestinal transplantation is required for these patients as a curative treatment, but immunosuppression and infections have had problems with transplantation and led to failure in early attempt^10^. It is typically restricted to patients suffering from life-threatening complications of parenteral or intravenous nutrition because of severe complications after transplantation, such as sepsis, chronic renal disease, and lymphoproliferative disease. Additionally, given the large content of lymphatic tissue in the graft, graft-versus-host disease is more frequent after intestinal transplantation than after transplantation of other organs^11^. Therefore, the overall 1- and 5-year survival rates have been reported to be 60 and 50%, respectively^12^^-^ 14, and intestinal transplantation remains more challenging than other types of organ transplantation.

Thus, HIOs have recently attracted attention as therapeutic cell sources. Since Sato et al. established an in-vitro three-dimensional culture system with LGR5+ intestinal epithelial stem cells in intestinal organoids^15^ various culture techniques for intestinal organoids have been reported^16^^,^ 17. However, there are still various problems with clinical application in humans because organoids do not exhibit the functional coordination between all germ layer-derived cells that develops during in-vivo organogenesis. Current human epithelial organoids are initially induced from the endoderm with a variety of growth factors, simultaneously suppress the formation of other germ layers^15^^,^ 16. Additionally, the small size and cystic shape of HIOs are ongoing challenges in need of resolution^18^.

Therefore, we focused on fetal pig intestines as xenografts and cell sources and compared the degree of in-vivo maturation after transplantation of spheroids versus fetal segmental tissues. Pigs, with their similarities in organ size, anatomy, and physiology to those of humans, are widely used in transplant research. The large supply of pigs is also an advantage when using fetal pig intestines as xenografts or cell sources. In addition, fetal organs have been reported to have an immunological advantage compared to adult differentiated organs when transplanted, especially in the renal setting^6^, so there is a possibility that fetal intestinal tissue could be used to construct an intestinal tract in humans while avoiding an immune response.

Our findings revealed the superiority of maturation in fetal intestinal tissue over that in spheroids. Maturation of the mucosa, smooth muscle, ganglion cells and nerve fibers were observed in transplanted fetal intestinal tissue, indicating that fetal segmental intestine can be developed and matured in vivo without artificial manipulations such as growth factor treatment, unlike embryonic stem cells or induced pluripotent stem cells. Furthermore, fetal pig organs grew larger in size after eight weeks, which might be an advantage when considering materials to use as grafts for transplantation. Meanwhile, the spheroids did not mature or develop in vivo, although they originally retained all the components related to structural maturation. We speculate that, once the tissues were disassembled enzymatically to generate single cells, interactions between neighboring cells were lost, and the rearrangement of the cells was disturbed. These results suggest that segmental fetal intestines might be more suitable than spheroids as grafts for xenotransplantation.

For the purpose of comparison with xenotransplantation in developmental size and maturation, syngeneic transplantation was also performed in C57BL/6JJmsSlc mice in this study. The syngeneic transplanted grafts showed sufficient maturation and grew larger than the pig grafts transplanted to immunodeficient mice four weeks after transplantation. We speculate that there is a difference in the speed of maturation between the animal species, since the gestational period of pigs is 114 days, while that of mice is 20 days. Additionally, a difference in growth rate between syngeneic and xenogeneic transplants might contribute, but it is difficult to explain the exact mechanism of the difference in growth rate due to species differences. Some growth hormones have been reported to be species-specific, while others have not, and the ways in which foreign hormones work in a xenogeneic situation are not fully understood^19^.

Regarding the transplantation site in the host animal, subrenal capsule and omental transplantation were performed in this study, and no obvious difference was observed in maturation. Many previous reports have shown that fetal rat intestines transplanted into the subcutaneous tissue, omentum, and renal capsule in adult rats can become vascularized and mature both morphologically and functionally^20^^-^ 23. For omental transplantation, there is an advantage in terms of the feasibility of transplantation and anastomosis with the host intestine after development because it is easy to simply wrap and roll small specimens such as fetal intestinal tissues; in addition, the grafts can mature and develop in a location quite close to the intestinal tract. On the other hand, there is a disadvantage: a risk that the specimen will migrate and exit the omentum due to body movement after transplantation, especially when the specimen is small. Subrenal capsule transplantation might be more suitable for small grafts such as spheroids.

The present study has several limitations. We were not able to verify the immunological advantage of the fetal intestine, since the development and maturation after in-vivo xenotransplantation were the main focuses of this study. Although Yang et al. revealed a lack of graft-versus-host disease after fetal intestine transplantation and suggested the potential application of fetal intestinal tissue as a graft for transplantation^24^, there have been few objective evaluations of the fetal intestinal immunological advantage, which was previously reported in the fetal renal field^6^^,^ 25. Thus, further investigations are needed, such as investigations into the abundance of antigen-presenting cells or the donor antigens such as major histocompatibility complex in fetal pig intestines. In addition, although maturation of the enteric nerve system was observed after transplantation, it was not possible to confirm rhythmic peristaltic movement in this study, while Nagy et al. observed spontaneous peristaltic activity in fetal human intestinal grafts transplanted into immunodeficient mice^26^. More quantitative and neurophysiological analyses of motility are needed to confirm the peristaltic wave in order to evaluate the development of the normal enteric nervous system.

Although there have been some problems with fetal intestinal xenotransplantation, the superiority of maturation and development achieved with segmental tissues versus spheroids was revealed in the study. The intra-abdominal fetal intestinal xenografts used in this model will be valuable platforms for the creation of intestinal grafts with immunological advantage.

## Conclusion

The structural maturation and development of fetal pig segmental intestinal tissues in mice were revealed, which may provide a platform for constructing intestinal grafts. In contrast, spheroids generated from fetal intestines did not mature in vivo after transplantation into mice.

## Data Availability

The data will be available upon request.
